# Long-term chronic infection of a young immunocompromised patient by the SARS-CoV-2 P.2 VOI

**DOI:** 10.1590/S1678-9946202466069

**Published:** 2024-12-06

**Authors:** Camila Malta Romano, Vitor Gabriel Lopes da Silva, Luciane Sussuchi da Silva, Carolina Sanchez Aranda, Cristina Mendes de Oliveira, Marilda Mendonça Teixeira Siqueira, Elisa Cavalcante Pereira, Paola Cristina Resende, Nancy Cristina Junqueira Bellei, José Eduardo Levi, Maria Isabel de Moraes-Pinto

**Affiliations:** 1 Universidade de São Paulo, Faculdade de Medicina, Instituto de Medicina Tropical de São Paulo, Laboratório de Virologia (LIM-52), São Paulo, São Paulo, Brazil; 2 Universidade Federal de São Paulo, Departamento de Pediatria, São Paulo, São Paulo, Brazil; 3 Dasa, São Paulo, São Paulo, Brazil; 4 Fiocruz, Instituto Oswaldo Cruz, Laboratório de Virus Respiratórios, Exantemáticos, Enterovírus e Emergências Virais, Rio de Janeiro, Rio de Janeiro, Brazil; 5 Universidade Federal de São Paulo, Laboratório de Virologia Clínica, São Paulo, São Paulo, Brazil

**Keywords:** Sars-CoV-2, Persistence, Immunocompromised, Intra-host evolution

## Abstract

An immunocompromised patient was infected by the SARS-CoV-2 variant of interest named Zeta (P.2) in February 2021. More than one year later, he suffered from symptomatic COVID-19 and sequencing revealed the same variant, which accumulated 23 substitutions. This case illustrates intra-host evolution of a particular SARS-CoV-2 variant, highlighting the importance of genomic surveillance of immunocompromised patients.

## INTRODUCTION

Numerous variants of severe acute respiratory syndrome coronavirus 2 (SARS-CoV-2), the causative agent of coronavirus disease 2019 (COVID-19), exist worldwide. Since its emergence, subsequent waves of COVID-19 have primarily been driven by the emergency of different variants with either increased transmissibility or the capacity to evade human immune responses^1^. Chronic SARS-CoV-2 infections in immunocompromised individuals is considered the most frequent mechanism for the emergence of genetically diverse SARS-CoV-2 variants, in which the persistence of the infection have been linked to intra-host viral evolution and immune evasion^2^. However, there is limited data on the genetic diversity of the virus in these long-term infections. Moreover, there are no current infection control guidelines tailored for immunocompromised individuals. In this study, we describe an immunocompromised patient with persistent SARS-CoV-2 infection for 431 days, whose the virus accumulated a number mutations consistent with intra-host evolution.

## CASE REPORT

The case described is from a 17-year-old Brazilian male, diagnosed at the age of six with X-linked agammaglobulinemia, on regular intravenous immunoglobulin (IVIg) replacement therapy. He showed a deletion (DelE407) in the TK domain of BTK. Baseline weight and height were 66.0 kg and 172 cm, respectively (body-mass index, BMI, 22.3).

Patient presented with a confirmed SARS-CoV-2 infection diagnosed on December 20^th^, 2020, initiated with flu-like symptoms, evolving into a deterioration of overall health. On January 24^th^, 2021, due to a 4-day progression marked by persistent fever, diarrhea, and generalized weakness, the patient was admitted to a university hospital in Sao Paulo State for further evaluation. During hospitalization, the patient needed oxygen supply but mechanical ventilation was never required. Moreover, high dose IVIg (1g/kg every 15-days), systemic (Prednisone, 60mg/day) and inhaled corticosteroid were administered. Patient was also treated with antimicrobial drugs. On day 50, high D-dimer levels were observed and computed tomography angiography diagnosed pulmonary thromboembolism and pulmonary infarction. He was treated for the condition, but high inflammatory markers and cytopenia persisted for a prolonged period.

After 77 days from the first episode, SARS-CoV-2 RT-PCR became negative with two subsequent negative tests recorded on March 29^th^, and April 7^th^, 2021. However, fever and cough persisted.

**Figure 1:**
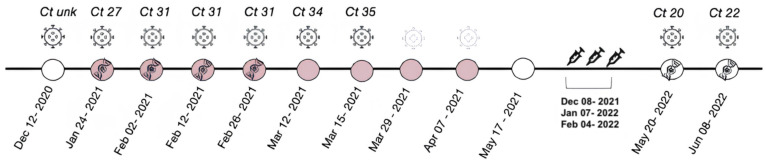
Timeline of key clinical and laboratory events, Including the dates of the RT-PCR tests, along with their corresponding Ct values. The periods during which the patient was hospitalized are represented in light pink. Negative RT-PCR tests are indicated by light-colored viral particles. The dates of viral genome sequencing are represented by a DNA symbol. The patient received three doses of the Pfizer-BioNTech BNT162b2 vaccine, starting in December 2021 (syringe symbol). *Ct unk = no information regarding the cycle threshold.

Patient gradually improved from oxygen need. On May 17^th^, 2021, after 10 days without oxygen supply, he was discharged from hospital, albeit still presenting with fever. At the time of discharge, the patient weighed 57 kg (BMI = 19.3). The patient received three doses of the Pfizer-BioNTech BNT162b2 vaccine, administered at 30-day intervals in December 2021, January 2022, and February 2022. Figure 1 illustrates the timeline of events, including viral load, hospitalization, and discharge, as well as information such as the dates of immunization and viral genome sequencing for both episodes.

In May 2022, the patient presented with daily fever for more than 30 days and mild malaise, but remained in generally good condition. Respiratory pattern remained stable, and a chest CT scan showed no new abnormalities. The patient tested positive for SARS-CoV-2 again in May and June 2022, with a RT-PCR cycle threshold (Ct) recorded in both episodes (Ct 20 and Ct 22, respectively) (Figure 1). During the follow-up, there was a progressive reduction of inflammatory markers accompanied by a gradual yet continuous weight gain. By January 2023, the patient's weight returned to 66.0 kg, coinciding with the resolution of fever.

This study was approved by the Brazilian National Research Ethics Committee (51535921.2.0000.5505) and the patient signed an informed consent form. The viral genetic data accessed in this study is registered under the number AAE5985 in the Brazilian National System for the Management of Genetic Heritage and Associated Traditional Knowledge (SisGen).

## MATERIALS AND METHODS

In total, six naso-oropharyngeal swabs for SARS-CoV-2 RT-PCR testing were collected at different times during the prolonged infection and sent to the central laboratory of Dasa in Barueri city, Sao Paulo State, Brazil (February 2021 and June 2022 samples) and/or to the Laboratorio de Virus Respiratorios, Exantematicos, Enterovirus e Emergencias Virais (LVRE – Laboratory of Respiratory Viruses, Exanthematics, Enteroviruses and Viral Emergencies), a Brazilian national reference laboratory, part of Oswaldo Cruz Institute (IOC), Fiocruz Rio de Janeiro (9 samples) for SARS-CoV-2 whole genome sequencing.

Viral genomes were sequenced using the ARTIC 4.1v primer set in the NovaSeq 6000 platform (Illumina, CA, USA) and assembled using the reference genome MN985325.1. Consensus FASTA sequences with more than 65% coverage were submitted to the GISAID^3^ database (Supplementary Table S1).

Although complete or partial sequences were obtained for six time points, only three SARS-CoV2 genomes had coverage >80% and were included in the phylogenetic analysis (February 12^th^, 2021; May 20^th^ and June 8^th^, 2022). These sequences were aligned to 80 reference genomes using the MAFFT multiple sequence alignment software version v7.407^4^ (see Supplementary Table S1 for IDs) and a maximum likelihood (ML) tree was reconstructed using the IQTREE2^5^ software employing the GTR+F+I substitution model. The TempEst software v.1.5.369^6^ identified samples with inconsistent temporal signal by regressing the genetic distances against sampling dates.

## RESULTS AND DISCUSSION

According to Pangolin^7^, all SARS-CoV-2 genomes obtained from the immunocompromised patient belong to the extinct lineage P.2 (alias for B.1.1.28.2 and considered a Variant of Interest (VOI) by World Health Organization when it emerged in Brazil in late 2020^8^). Since SARS-CoV-2 genomes sampled during the acute infection mirror the contemporaneous circulating lineages, the hypothesis of reinfection in 2022 by P.2 is unlikely, as in the latter half of 2021, the P.2 lineage as well as other B.1.1.28-variants were superseded by the Delta lineage, and then by Omicron. Since early 2022, only Omicron lineages were detected in the country^9^.

**Figure 2:**
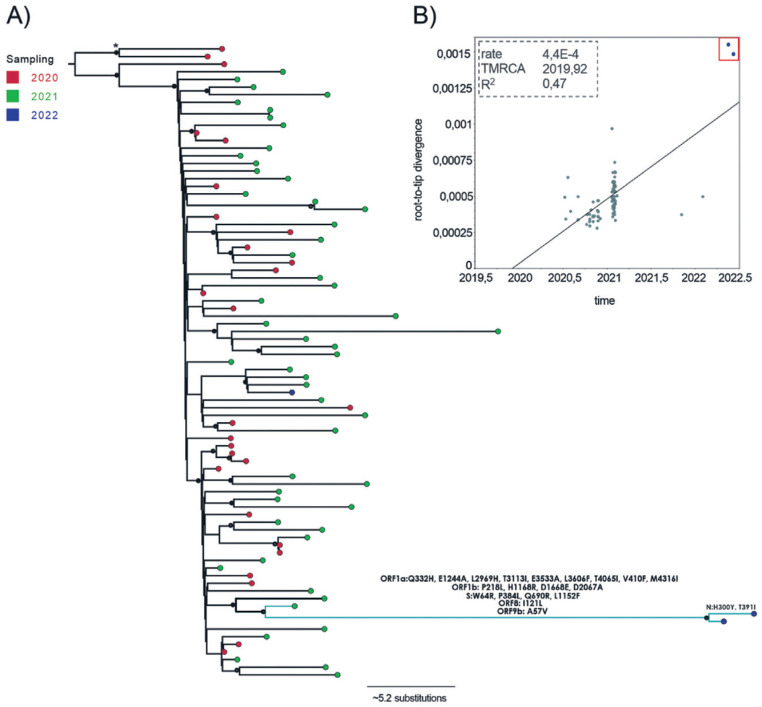
Evolutionary analysis of the SARS-CoV-2 lineage P.2.: (A) Maximum likelihood phylogenetic tree including 81 reference sequences from Brazil and other countries (78 from P.2 variant and 2 from B.1.28 lineage as outgroup). Samples/taxa were colored according to their collection year (2020 in red, 2021 in green, and 2022 in violet). Asterisk represents the outgroup. The branch leading to the 2021 and 2022 sequences from the patient from this study are highlighted in light blue. Black dots were positioned in nodes with more than 90% bootstrap support; (B) Root-to-tip plot showing the regression of genetic distance against time. Samples from 2022 from this study are outliers and are indicated by the red box in the up-right corner.

To completely rule out the possibility of reinfection, a ML tree was reconstructed using the three genomes obtained from the immunocompromised patient (>90% coverage) and representative P.2 genomes from Brazil and elsewhere. According to the tree (Figure 2A), the patient's SARS-CoV-2 genomes from 2021 and 2022 clustered together in a well-supported clade (>90% bootstrap), close to sequences from the beginning of 2021.

The root-to-tip analysis showed a moderate association between genetic distance and sampling dates (R^2^ = 0.47). Notably, the genetic divergence of the two genomes from 2022 was strongly incongruent with their sampling date, indicative of a substantial excess of nucleotide substitution (Figure 2B). The excess of mutations in viruses from chronic infection have been already reported, in which the within-host substitution rates can be ∼2 times higher than the global evolutionary rate, enabling the emergence of distinct lineages in chronic infected patients^1,10,11^.

**Figure 3:**
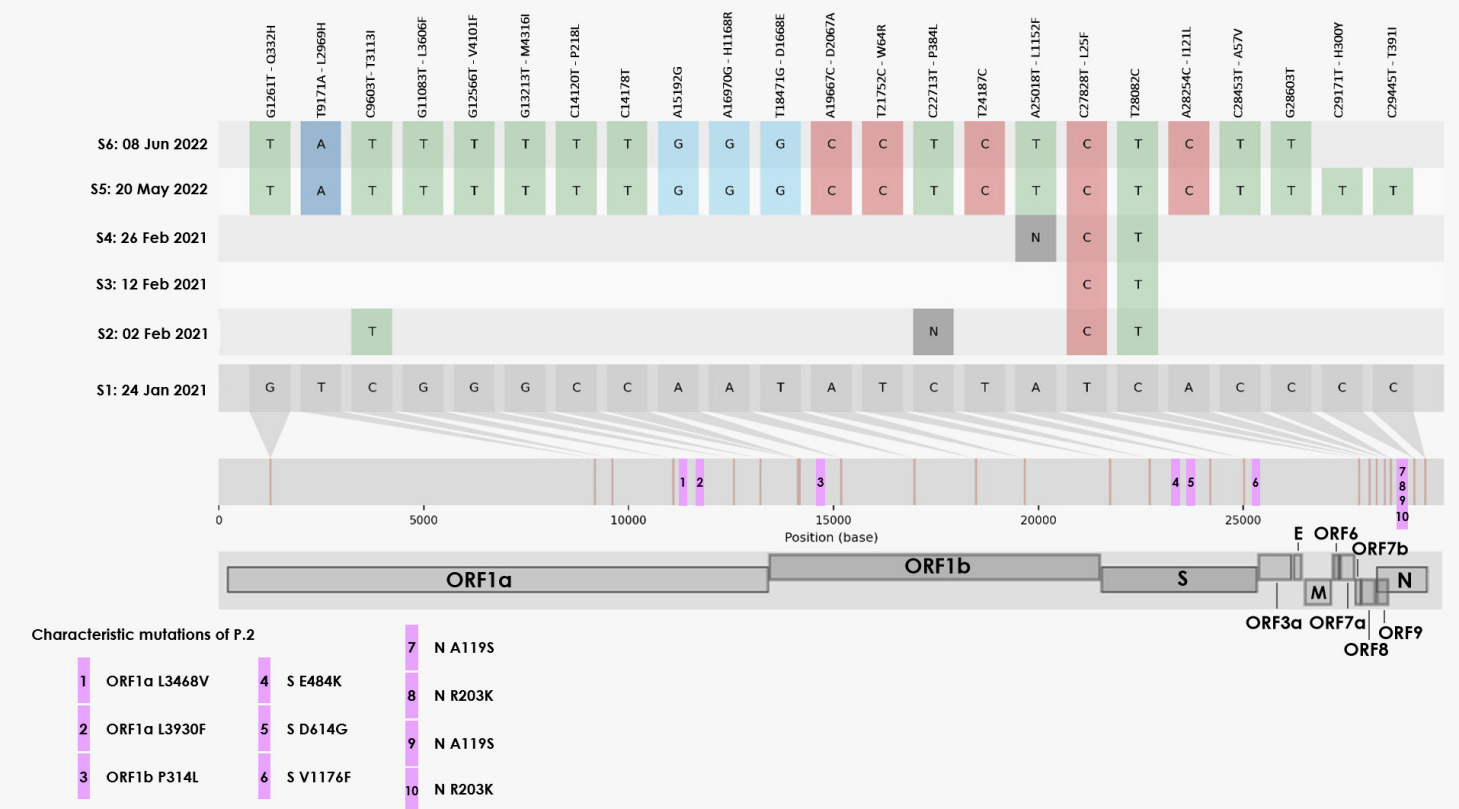
Analysis of intra-host single nucleotide variations (iSNVs) in samples from the patient infected by the P.2 lineage. S1 to S6 refer to the samples/collection dates from January 24, 2021 until June 8, 2022. The signature mutations of P2 lineages are in purple, while the specific changes occurred intra-host are depicted in the particular sample (S1-S6) and colored accordingly. The P.2 lineage used as reference was EPI_ISL_2777369.

The rapid evolution of these viruses led them to accumulate 23 substitutions in 1.3 years (see Figure 2A and Figure 3 for unique mutations in each genome). The highest frequency of mutations occurred from nucleotide C-T (39%) as observed elsewhere^12^. In total, 78% (18/23) of the mutations shared by the May and June, 2022 viruses were non-synonymous, which is close to the expected if both synonymous and non-synonymous mutations occurred in the absence of transmission bottlenecks^13^. However, the distribution of these mutations was not random, with a higher proportion of changes occurring in structural genes (N, E, and S) in comparison to the NS genes in ORF1ab (0.4% and 0.07%, respectively). Notably, four substitutions were observed within the spike protein in samples S5 and S6, S:W64R, P364L, Q690R, and L1152F (Figure 3). This is consistent with immune system selection pressures (particularly cellular) against surface proteins. As far as we know, the substitutions in the Spike of the 2022 viruses were not previously associated with immune evasion or higher infectivity, though some of them were also described in viruses from chronically infected patients, such as W64 in the S1 NTD domain^1,11^ and P384L in the RDB^1^. We highlight that none of these mutations were observed in reads from the 2021 viruses. Among the 23 mutated sites, only four were present as minor variants from the 2021 sample; three in ORF1ab and one in the envelope gene (ET30I). The ET30I site is located at the transmembrane domain and was detected in 36% of the reads from the first sample. Unfortunately, no intermediate samples (from February 2021 to May 2022) were available for a deeper understanding of intra-host viral evolution.

To the best of our knowledge, this is the longest case of Zeta (P.2) SARS-CoV-2 persistence described to date (431 days). Previously documented long-term infections lasted 471 days, involving a B.1.517 lineage in an immunocompromised individual with lymphocytic leukemia and B cell lymphoma^1^, and 486 days, featuring a B.1.2 lineage in an immunocompromised individual with common variable immunodeficiency following monotherapy with the monoclonal antibody Bamlanivimab^14^.

## CONCLUSION

This case underscores that immunocompromised individuals can harbor SARS-CoV-2 for extended periods, during which accelerated evolution may occur under a weak selective pressure imposed by compromised immune responses. The low Ct value from the June 2022 sample suggests active excretion and potential for transmission. The resurgence of an ancient SARS-CoV-2 with newly acquired mutations may be of risk for individuals unexposed to these lineages, such as newborns and those starting their immunization with current Omicron monovalent vaccines.

## References

[1] Chaguza C, Hahn AM, Petrone ME, Zhou S, Fergunson D, Breban MI (2023). Accelerated SARS-CoV-2 intrahost evolution leading to distinct genotypes during chronic infection. Cell Rep Med.

[2] Weigang S, Fuchs J, Zimmer G, Schnepf D, Kern L, Beer J (2021). Within-host evolution of SARS-CoV-2 in an immunosuppressed COVID-19 patient as a source of immune escape variants. Nat Commun.

[3] Khare S, Gurry C, Freitas L, Schultz MB, Bach G, Diallo A (2021). GISAID's role in pandemic response. China CDC Wkly.

[4] Katoh K, Standley DM (2013). MAFFT Multiple Sequence Alignment Software Version 7: Improvements in performance and usability. Mol Biol Evol.

[5] Minh BQ, Schmidt HA, Chernomor O, Schrempf D, Woodhams MD, Haeseler A (2020). IQ-TREE 2: new models and efficient methods for phylogenetic inference in the genomic era. Mol Biol Evol.

[6] Rambaut A, Lam TT, Carvalho LM, Pybus OG (2016). Exploring the temporal structure of heterochronous sequences using TempEst (formerly Path-O-Gen). Virus Evol.

[7] Pangolin COVID-19 lineage assigner.

[8] Voloch CM, Francisco R, Almeida LG, Cardoso CC, Brustolini OJ, Gerber AL (2021). Genomic characterization of a novel SARS-CoV-2 lineage from Rio de Janeiro, Brazil. J Virol.

[9] Fundação Oswaldo Cruz Rede Genômica Fiocruz.

[10] Choudhary MC, Crain CR, Qiu X, Hanage W, Li JZ (2022). Severe acute respiratory syndrome Coronavirus 2 (SARS-CoV-2) sequence characteristics of Coronavirus Disease 2019 (COVID-19) persistence and reinfection. Clin Infect Dis.

[11] Kemp SA, Collier DA, Datir RP, Ferreira IA, Gayed S, Jahun A (2021). SARS-CoV-2 evolution during treatment of chronic infection. Nature.

[12] Tonkin-Hill G, Martincorena I, Amato R, Lawson AR, Gerstung M, Johnston I (2021). Patterns of within-host genetic diversity in SARS-CoV-2. Elife.

[13] Holmes EC (2003). Patterns of intra- and interhost nonsynonymous variation reveal strong purifying selection in Dengue virus. J Virol.

[14] Halfmann PJ, Minor NR, Haddock III LA, Maddox R, Moreno GK, Braun KM (2023). Evolution of a globally unique SARS-CoV-2 Spike E484T monoclonal antibody escape mutation in a persistently infected, immunocompromised individual. Virus Evol.

