# Supplementary material for: Long-term chronic infection of a young immunocompromised patient by the SARS-CoV-2 P.2 VOI
Source: Rev Inst Med Trop Sao Paulo. 2024 Dec 6;66:e69. doi: 10.1590/S1678-9946202466069 (PMC11654138; doi:10.1590/S1678-9946202466069)
Supplement: Supplementary file 1 [file 1678-9946-rimtsp-66-S1678-9946202466069suppl01.pdf]

REVISTA DO INSTITUTO DE MEDICINA TROPICAL DE SÃO PAULO
JOURNAL OF THE SÃO PAULO INSTITUTE OF TROPICAL MEDICINE

SUPPLEMENTARY MATERIAL

https://doi.org/10.48331/scielodata.EI0XQT

Long-term chronic infection of a young immunocompromised patient by the SARS-CoV-2 P.2 VOI

Camila Malta Romano [1*], Vitor Gabriel Lopes da Silva [2*], Luciane Sussuchi da Silva[3], Carolina Sanchez Aranda [2], Cristina Mendes de Oliveira[3], Marilda Mendonça Teixeira Siqueira[4], Elisa Cavalcante Pereira[4], Paola Cristina Resende[4], Nancy Cristina Junqueira Bellei[5], José Eduardo Levi [1,3], Maria Isabel de Moraes-Pinto [2,3]

[1]Universidade de São Paulo, Faculdade de Medicina, Instituto de Medicina Tropical de São Paulo, Laboratório de Virologia (LIM-52), São Paulo, São Paulo, Brazil

[2]Universidade Federal de São Paulo, Departamento de Pediatria, São Paulo, São Paulo, Brazil

[3]Dasa, São Paulo, São Paulo, Brazil

[4]Fiocruz, Instituto Oswaldo Cruz, Laboratório de Virus Respiratórios, Exantemáticos, Enterovírus e Emergências Virais, Rio de Janeiro, Rio de Janeiro, Brazil

[5]Universidade Federal de São Paulo, Laboratório de Virologia Clínica, São Paulo, São Paulo, Brazil

*These authors contributed equally to the article

Correspondence to: José Eduardo Levi Dasa, Avenida Juruá, 548, Alphaville, CEP 06455-010, Barueri, SP, Brazil

E-mail: jose.levi@dasa.com.br

Received: 29 June 2024

Accepted: 30 October 2024

Supplementary Table S1 - GISAID ID and metadata from samples used in the analysis. Samples from this study are colored in red

GISAID ID	Lineage	Origin	State	Sampling year
B1_1_28_hCoV-19_Brazil_RJ-00414_2020_EPI_ISL_717816_2020-09-03	B1	Brazil	RJ	2020
B1_1_28_hCoV-19_Brazil_RJ-00417_2020_EPI_ISL_717819_2020-10-09	B1	Brazil	RJ	2020
hCoV-19/Brazil/un-NVBS25133GENOV7587EPI_ISL_15856103_2022-06-08	P2	Brazil	SP	2022
hCoV-19_Brazil_SP-FIOCRUZ-20261_2022_EPI_ISL_15436498_2022-05-20	P2	Brazil	SP	2022
hCoV-19/Brazil/un-NVBS25132GENOV7587_EPI_ISL_15856102_2021-02-12	P2	Brazil	SP	2021
**hCoV-19/Brazil/SP-FIOCRUZ-68993/2021_ EPI_ISL_18739166_2021_01_24	P2	Brazil	SP	2021
**hCoV-19/Brazil/SP-FIOCRUZ-68994/2021_EPI_ISL_18739167_2021_02_02	P2	Brazil	SP	2021
**hCoV-19/Brazil/SP-FIOCRUZ-68996/2021_EPI_ISL_18739168_2021_02_26	P2	Brazil	SP	2021
hCoV-19_Argentina_PAIS-A0452_2021_EPI_ISL_1395782_2021-02-05	P2	Argentina	BA	2021
hCoV-19_Bolivia_SAN-UPCH-1044_2020_EPI_ISL_5147887_2020-09-03	P2	Bolivia	Sant	2020
hCoV-19_Brazil_AL-FIOCRUZ-28256_2020_EPI_ISL_792639_2020-11-21	P2	Brazil	BR_other	2020
hCoV-19_Brazil_AL-FIOCRUZ-30270_2020_EPI_ISL_792642_2020-12-04	P2	Brazil	BR_other	2020
hCoV-19_Brazil_BA-00737_2021_EPI_ISL_1213386_2021-01-24	P2	Brazil	BR_other	2021
hCoV-19_Brazil_BA-FIOCRUZ-PVM48578_2021_EPI_ISL_13859929_2021-01-22	P2	Brazil	BR_other	2021
hCoV-19_Brazil_BA-FIOCRUZ-PVM86772_2021_EPI_ISL_6908710_2021-01-29	P2	Brazil	BR_other	2021
hCoV-19_Brazil_CE-FIOCRUZ-00696_2021_EPI_ISL_3912472_2021-01-23	P2	Brazil	BR_other	2021
hCoV-19_Brazil_CE-FIOCRUZ-727770-237-2_2021_EPI_ISL_2801372_2021-02-01	P2	Brazil	BR_other	2021
hCoV-19_Brazil_DF-1028R1_2020_EPI_ISL_882657_2020-07-23	P2	Brazil	BR_other	2020

This is an open-access article distributed under the terms of the Creative Commons Attribution License.

Supplementary Table S1 - GISAID ID and metadata from samples used in the analysis. Samples from this study are colored in red. (cont.)

GISAID ID	Lineage	Origin	State	Sampling year
hCoV-19_Brazil_ES-FIOCRUZ-3582_2021_EPI_ISL_2645582_2021-01-22	P2	Brazil	BR_other	2021
hCoV-19_Brazil_ES-FIOCRUZ-3603_2021_EPI_ISL_2645585_2021-01-24	P2	Brazil	BR_other	2021
hCoV-19_Brazil_ES-LACENES-320169627_2022_EPI_ISL_13833729_2022-02-02	P2	Brazil	BR_other	2022
hCoV-19_Brazil_MG-FUNED-02-21_2021_EPI_ISL_1182555_2021-01-30	P2	Brazil	BR_other	2021
hCoV-19_Brazil_MG-LBI56_2021_EPI_ISL_1494964_2021-01-25	P2	Brazil	BR_other	2021
hCoV-19_Brazil_MG-LBI57_2021_EPI_ISL_1494965_2021-01-27	P2	Brazil	BR_other	2021
hCoV-19_Brazil_MS-GD144_2021_EPI_ISL_12920688_2021-01-27	P2	Brazil	BR_other	2021
hCoV-19_Brazil_MS-GD15_2021_EPI_ISL_8975223_2021-01-22	P2	Brazil	BR_other	2021
hCoV-19_Brazil_MS-HRMS_1345_2020_EPI_ISL_6633660_2020-08-03	P2	Brazil	BR_other	2020
hCoV-19_Brazil_MS-HRMS_1617_2020_EPI_ISL_6633694_2020-07-10	P2	Brazil	BR_other	2020
hCoV-19_Brazil_PB-FIOCRUZ-29647_2020_EPI_ISL_792635_2020-11-10	P2	Brazil	BR_other	2020
hCoV-19_Brazil_PR-FIOCRUZ-28601_2020_EPI_ISL_792645_2020-10-27	P2	Brazil	BR_other	2020
hCoV-19_Brazil_PR-FIOCRUZ-28602_2020_EPI_ISL_792646_2020-11-03	P2	Brazil	BR_other	2020
hCoV-19_Brazil_RJ-00571_2020_EPI_ISL_717955_2020-11-05	P2	Brazil	BR_other	2020
hCoV-19_Brazil_RJ-00572_2020_EPI_ISL_717956_2020-11-05	P2	Brazil	BR_other	2020
hCoV-19_Brazil_RJ-00573_2020_EPI_ISL_717957_2020-11-05	P2	Brazil	BR_other	2020
hCoV-19_Brazil_RJ-00812_2021_EPI_ISL_1213374_2021-01-25	P2	Brazil	BR_other	2021
hCoV-19_Brazil_RJ-00819_2021_EPI_ISL_1213378_2021-01-25	P2	Brazil	BR_other	2021
hCoV-19_Brazil_RJ-FIOCRUZ-00310_2021_EPI_ISL_15143063_2021-11-06	P2	Brazil	BR_other	2021
hCoV-19_Brazil_RJ-FIOCRUZ-4192_2021_EPI_ISL_2614193_2021-02-01	P2	Brazil	BR_other	2021
hCoV-19_Brazil_RJ-FIOCRUZ-4316_2021_EPI_ISL_2614194_2021-02-01	P2	Brazil	BR_other	2021
hCoV-19_Brazil_RJ-FIOCRUZ-4317_2021_EPI_ISL_2614195_2021-02-01	P2	Brazil	BR_other	2021
hCoV-19_Brazil_RJ-FUNED-330256870_2021_EPI_ISL_1182552_2021-02-02	P2	Brazil	BR_other	2021
hCoV-19_Brazil_RS-00604_2020_EPI_ISL_770556_2020-11-23	P2	Brazil	BR_other	2020
hCoV-19_Brazil_RS-00628_2020_EPI_ISL_770553_2020-11-24	P2	Brazil	BR_other	2020
hCoV-19_Brazil_RS-00649_2020_EPI_ISL_770595_2020-11-25	P2	Brazil	BR_other	2020
hCoV-19_Brazil_RS-00650_2020_EPI_ISL_770596_2020-11-25	P2	Brazil	BR_other	2020
hCoV-19_Brazil_RS-00653_2020_EPI_ISL_770598_2020-11-24	P2	Brazil	BR_other	2020
hCoV-19_Brazil_RS-00659_2020_EPI_ISL_770602_2020-11-26	P2	Brazil	BR_other	2020
hCoV-19_Brazil_RS-00660_2020_EPI_ISL_770603_2020-11-26	P2	Brazil	BR_other	2020
hCoV-19_Brazil_RS-00682_2020_EPI_ISL_770554_2020-11-27	P2	Brazil	BR_other	2020
hCoV-19_Brazil_SC-FIOCRUZ-14556_2021_EPI_ISL_2677080_2021-01-25	P2	Brazil	BR_other	2021
hCoV-19_Brazil_SC-FIOCRUZ-7210_2021_EPI_ISL_2677110_2021-01-30	P2	Brazil	BR_other	2021
hCoV-19_Brazil_SC-UFSC-186_2021_EPI_ISL_7743978_2021-01-23	P2	Brazil	BR_other	2021
hCoV-19_Brazil_SP-1758_2021_EPI_ISL_1078997_2021-02-05	P2	Brazil	SP	2021
hCoV-19_Brazil_SP-1806_2021_EPI_ISL_1171651_2021-02-01	P2	Brazil	SP	2021
hCoV-19_Brazil_SP-1833_2021_EPI_ISL_1171673_2021-02-02	P2	Brazil	SP	2021
hCoV-19_Brazil_SP-1850_2021_EPI_ISL_1219024_2021-02-05	P2	Brazil	SP	2021
hCoV-19_Brazil_SP-1867_2021_EPI_ISL_1293068_2021-02-02	P2	Brazil	SP	2021
hCoV-19_Brazil_SP-2079_2021_EPI_ISL_1468456_2021-02-02	P2	Brazil	SP	2021
hCoV-19_Brazil_SP-2105_2021_EPI_ISL_1468467_2021-01-30	P2	Brazil	SP	2021
hCoV-19_Brazil_SP-2195_2021_EPI_ISL_1520113_2021-02-05	P2	Brazil	SP	2021
hCoV-19_Brazil_SP-2199_2021_EPI_ISL_1520115_2021-02-05	P2	Brazil	SP	2021
hCoV-19_Brazil_SP-BT13024_2021_EPI_ISL_1000677_2021-01-22	P2	Brazil	SP	2021
hCoV-19_Brazil_SP-FIOCRUZ-68905_2021_EPI_ISL_13822498_2021-01-29	P2	Brazil	SP	2021

Supplementary Table S1 - GISAID ID and metadata from samples used in the analysis. Samples from this study are colored in red. (cont.)

GISAID ID	Lineage	Origin	State	Sampling year
hCoV-19_Brazil_SP-IB_RP16465_2021_EPI_ISL_14204703_2021-02-04	P2	Brazil	SP	2021
hCoV-19_Brazil_SP-L23-CAMPI332_2020_EPI_ISL_13046402_2020-07-14	P2	Brazil	SP	2020
hCoV-19_Brazil_SP-SJRP187_2021_EPI_ISL_2544860_2021-01-26	P2	Brazil	SP	2021
hCoV-19_Brazil_SP-SJRP200_2021_EPI_ISL_2544863_2021-01-26	P2	Brazil	SP	2021
hCoV-19_Brazil_SP-SJRP207_2021_EPI_ISL_2544868_2021-01-26	P2	Brazil	SP	2021
hCoV-19_Brazil_SP-SJRP214_2021_EPI_ISL_2544871_2021-01-26	P2	Brazil	SP	2021
hCoV-19_England_RAND-12672B1_2021_EPI_ISL_1045717_2021-02-01	P2	England	England	2021
hCoV-19_Norway_2577_2021_EPI_ISL_1317876_2021-01-31	P2	Norway	Norway	2021
hCoV-19_Switzerland_BS-UHB-42496208_2020_EPI_ISL_1014691_2020-10-21	P2	Switz	Switz	2020
hCoV-19_USA_AZ-TG832207_2020_EPI_ISL_1909385_2020-10-22	P2	USA	AZ	2020
hCoV-19_USA_AZ-TG920815_2020_EPI_ISL_8328857_2020-10-22	P2	USA	AZ	2020
hCoV-19_USA_AZ-TG954977_2020_EPI_ISL_2919296_2020-10-21	P2	USA	AZ	2020
hCoV-19_USA_CA-CDPH-3000000191_2021_EPI_ISL_12978338_2021-02-01	P2	USA	CAL	2021
hCoV-19_USA_CA-CDPH-3000001277_2021_EPI_ISL_12977751_2021-02-01	P2	USA	CAL	2021
hCoV-19_USA_CA-CDPH-3000010814_2021_EPI_ISL_2882677_2021-02-05	P2	USA	CAL	2021
hCoV-19_USA_CA-CZB-26923_2021_EPI_ISL_1235114_2021-02-01	P2	USA	CAL	2021
hCoV-19_USA_FL-BPHL-0652_2021_EPI_ISL_1132187_2021-02-04	P2	USA	FLO	2021
hCoV-19_USA_IN-CDC-STM-000008492_2021_EPI_ISL_1016557_2021-02-01	P2	USA	IND	2021
hCoV-19_USA_MD-HP08842-PIDFYTQFGD_2020_EPI_ISL_3814035_2020-10-11	P2	USA	MD	2021
hCoV-19_USA_ME-HETL-J1381_2021_EPI_ISL_1048739_2021-02-03	P2	USA	ME	2021
hCoV-19_USA_OH-21AM-030CO00265_2021_EPI_ISL_2312842_2021-01-30	P2	USA	OHI	2021
hCoV-19_USA_TX-HMH-MCoV-26001_2021_EPI_ISL_1235781_2021-01-31	P2	USA	TEX	2021
hCoV-19_USA_VA-CAV_VAS3N_00000799_01_2021_EPI_ISL_1856671_2021-02	P2	USA	VAN	2021

**Sequences from the same patient but not used in phylogenetic analysis due to low horizontal coverage (<70%).
